# Prevalence of tobacco smoking in a sample of general secondary school students in Cairo and its correlation to other psychiatric disorders

**DOI:** 10.1186/s43045-022-00252-7

**Published:** 2022-10-13

**Authors:** Heba Hamed ElShahawi, Ghada Refaat Amin, Sherien Ahmed Khalil, Mahmoud Hassan Morsy, Mohamed Gamal Farwiez, Mayar Medhat Nawara

**Affiliations:** 1 Psychiatry Department, Faculty of Medicine, Ain Shams University, Cairo, Egypt; 2 Okasha Institute of Psychiatry, Ain Shams University, 22, Dair Al-Malak, Abbassia, Cairo, 11657 Egypt

**Keywords:** Tobacco, Smoking, Adolescents, Depression, Anxiety, Eating disorders, Impulsivity, Psychiatric disorders, Egypt

## Abstract

**Background:**

Tobacco smoking is considered currently a global public health concern among youth especially school students. There is a scarcity of data about the impact of this global phenomenon in Middle Eastern countries. This study assesses the prevalence of tobacco smoking, including conventional cigarettes, E-cigarettes, and water pipe and its sociodemographic and psychiatric correlates among a sample of general secondary school students in Cairo, Egypt.

**Results:**

A convenient sample of 391 Egyptian general secondary students from different areas of Greater Cairo were assessed using the Socioeconomic scale, Fagerstrom Test For Nicotine Dependence, The Lebanon Water pipe Dependence Scale, questionnaire for electronic cigarette use, Barratt impulsivity scale 11, and The MINI International Neuropsychiatric Interview for Children and Adolescents. 12.8% (*n* = 50) were smokers. There is a significant relation between family history of smoking and substance use and smoking and significant relation between smoking, impulsivity, and mood symptoms.

**Conclusions:**

Prevalence of tobacco smoking in Egyptian adolescents was 12.8% with significant relation between smoking and impulsivity and mood symptoms.

## Background

Tobacco smoking is widely spread worldwide with a prevalence of about 20.2% around the globe [1]*.* It is the only legalized drug that causes death for its users and people around, being responsible for 6 million deaths per year with 10 % of the deaths due to passive smoking [2]. It also is considered an important risk factor for many chronic diseases that cause 80% of premature mortality worldwide [3]; therefore, tobacco cessation is one of the important goals for prevention and control of chronic diseases, disability, and mortality [4]*.*

Tobacco use in its multiple forms has other negative mental health outcomes such as mood disorders and suicide [5, 6]***.*** Studies found co-morbidity of eating disorders [7], problematic internet use, gambling disorder, and sexual disorders [8] with nicotine dependence; it was also found that smoking is related to schizophrenia and ADHD [6, 9].

In recent years, many forms of tobacco products emerged as heated tobacco [10] and Electronic Nicotine Delivery Systems (ENDS) like electronic cigarettes (e-cigarettes) [11].

E-cigarette use, also known as vaping, is smoking inhalable aerosol from a nicotine-containing solution that is evaporated from a battery-operated device that is similar to tobacco smoke in taste and inhaling sensations. ENDS use is controversial in many aspects. It has been marketed with the potential for smoking reduction or cessation; however, much is still unknown about the consequences of its long-term use, the extent of carcinogens it has, and the role of these products in initiating and perpetuating nicotine dependence in teenagers and young adults [12].

Most of the world’s smokers come from low- and middle-income countries, with 80% of the annual tobacco-related mortality expected to occur in these countries by 2030 [13]. However, there is a scarcity of available data about the prevalence of tobacco smoking in adolescents in Egypt. The aim of this study was to investigate the prevalence of tobacco smoking among general secondary school students—including conventional cigarettes, E-cigarettes, and water pipe; associated socioeconomic risk factors; academic achievement; psychiatric comorbidities; and personality traits. We hypothesized that comorbidity with other psychiatric disorders is more prevalent in tobacco-using adolescents than in their non-tobacco-using peers.

## Methods

### Participants

A cross-sectional comparative study was conducted by conveniently recruiting secondary school students ages 15–17 years of both genders from the Greater Cairo area online via social media and self-administrated questionnaire from educational centers due to COVID-19 restrictions. Using PASS 11 program, based on the prevalence of cigarette smoking in secondary school students of (12.4% (±) 5) shown by [14], a sample size of 450 students was calculated with confidence level of 95%. Four hundred fifty secondary school students in the Greater Cairo area responded, 59 of which were excluded because they were studying other secondary stage certificates (International, Azhar, Vocational) and were not matching with the same age group. The collection of data took place between March 2021 and September 2021.

Confidentiality of collected data was ensured (i.e., no names, identification numbers, or any other personal identifiers were requested) so students could feel safe enough to share accurate information. All participants were asked to sign an informed consent explaining the purpose of the study, stating their approval to participate in the study, and ensuring confidentiality of their information.

The study was approved by Ain-Shams University research and ethical committee (FMASU REC FWA00017585) (FMASU MD85/2020) (SID:337). Data were stored on a password-protected computer in a locked office of the research team and access was strictly limited to study investigators.

After exclusion of ineligible participants, 391 subjects completed a questionnaire for sociodemographic data.

*The socioeconomic scale* [15] which is an Arabic version of a 7-item scale assesses the family’s socioeconomic status.

*Fagerstrom Test For Nicotine Dependence (FTND) to assess the severity of cigarette use* [16, 17]: an Arabic version of a standard instrument contains six items that evaluate the quantity of cigarette consumption, the compulsion to use, and dependence for assessing the intensity of physical addiction to nicotine.

*Lebanon Water-pipe Dependence Scale (LWDS-11)* [18]: an Arabic version of a scale that is formed of four subscales to assess the severity of water pipe use used for evaluation of water pipe dependence according to a cutoff score of 10.

*Questionnaire for electronic cigarette* [19]: an Arabic questionnaire to describe patterns and reasons for E-cigarette use whether it is social causes or due to dependence.

*Barratt impulsivity scale 11* [20–22]: an Arabic version of a 30-item scale to assess the severity of impulsivity for the subjects according to cutoff score of 10 in water pipe smokers. The scale consists of 11 items under 4 subscales assessing nicotine dependence, negative reinforcement, psychological craving, and positive reinforcement in Likert-type questions.

*MINI International Neuropsychiatric Interview for Children and Adolescents* (MINI-Kid) [23, 24]: an Arabic version of a short structured psychiatric interview to screen the psychiatric symptoms in subjects.

### Statistical analysis

The collected data was revised, coded, and tabulated using Statistical Package for Social Science (SPSS 25) (version 25).

Data was presented and suitable analysis was done according to the type of data obtained for each parameter.

Data was plot for normality using *Kolmogorov*–*Smirnov* test and the following tests were performed:

*Descriptive statistics showing* mean, standard deviation (± SD), and range for parametric numerical data, while median and interquartile range (IQR) for non-parametric numerical data and frequency and percentage of non-numerical data.

*Analytical statistics using* ***c****hi-square test* to examine the relationship between two qualitative variables and *Fisher’s exact test* was used to examine the relationship between two qualitative variables when the expected count is less than 5 in more than 20% of cells.

Level of significance using the *P-value*: *P* > 0.05, non-significant (NS); *P* < 0.05, significant (S).

## Results

### Participants’ characteristics

Our study group included 55.2% (*n* = 216) females and 44.8% (*n* = 175) males with mean age (16.65±0.55). 84.7% (*n* = 331) were of high socio-economic status, while 14.3% (*n* = 56) were of middle and 1% (*n* = 4) were of low socio-economic status; 7.4% (*n* = 29) of the study group had family history of substance use while 49.6% (*n* = 194) had family history of smoking and there was significant relation between family history of substance use (*p* = 0.001), family history of smoking (*χ*^*2*^***=*** 13.636, *p* < 0.001), and smoking in adolescents.

### Impulsivity

Barratt impulsivity scale-11 showed average score on impulsivity for the study group (62.49 ± 9.52). Impulsivity was significantly related with smoking as shown in Table 1, there was a significant relation between electronic cigarette smoking in adolescent smokers and impulsivity versus other types of smoking as shown in Table 2, also, there was no significant relation between gender difference and impulsivity in smokers (*t =* 0.706, *p* = 0.483), and there was no significant relation between pattern of smoking and impulsivity (*p* = 0.281, *p* = 0.323).

**Table 1:** Gender and socioeconomic status differences in adolescent smokers

Smokers group		Gender		Chi-square test	Socioeconomic status		Fisher’s exact test
		Male	Female		High *N* (%)	Low *N* (%)	
		*N* (%)	*N* (%)	***χ***^***2***^ *p*-value Sig.			*p*-value Sig.
cig smoking	No	14 (51.85%)	14 (60.87%)	0.41 0.522 (NS)	21 (53.85%)	7 (63.64%)	0.734 (NS)
	Yes	13 (48.15%)	9 (39.13%)		18 (46.15%)	4 (36.36%)	
E-cig smoking	No	8 (29.63%)	4 (17.39%)	1.02 0.313 (NS)	10 (25.64%)	2 (18.18%)	1.00 (NS)
	Yes	19 (70.37%)	19 (82.61%)		29 (74.36%)	9 (81.82%)	
Water pipe smoking	No	5 (18.52%)	11 (47.83%)	4.903 0.027 (S)	13 (33.33%)	3 (27.27%)	1.00 (NS)
	Yes	22 (81.48%)	12 (52.17%)		26 (66.67%)	8 (72.73%)	

*Sig.*, significant; *S*, significant; *NS*, non-significant

**Table 2:** Impulsivity in adolescent smokers according to Barratt impulsivity scale 11

		Smoker		Test of significance		
		Non-smoker (*n* = 341)	Smoker (*n* = 50)			
		Mean ± SD *N* (%)	Mean ± SD *N* (%)	Value	*p*-value	Sig.
Impulsivity	Total impulsivity	63.93 ± 7.99	52.43 ± 12.84	***t =*** 6.103	<0.001	S
	Mild	276 (80.94%)	40 (80%)	***χ***^***2***^ ***=*** 0.485	0.785	NS
	Moderate	57 (16.72%)	8 (16%)			
	Severe	8 (2.35%)	2 (4%)			

*t*, Student *t*-test of significance; *χ*^*2*^, chi-square test of significance

### Psychiatric symptoms

Our study found a significant relation between mood symptoms as depressive and manic symptoms and smoking as shown in Table 3; also there was a significant relation between patterns of smoking and depressive symptoms (***X***^***2***^= 8.923, *p* = 0.012); also there was a significant relation between cigarette smoking in particular and depressive symptoms, manic symptoms, suicidal ideations, panic symptoms, and binge eating symptoms as shown in Table 4 while there was no significance between electronic cigarette smoking and water pipe smoking and psychiatric symptoms; and also there was a significant relation between gender of the smoker and psychiatric symptoms in the form of panic symptoms (*χ*^*2*^ = 4.78, *p* = 0.029).

**Table 3:** Impulsivity in adolescents using different forms of tobacco according to Barratt impulsivity scale 11

		Total impulsivity	Mild	Moderate	Severe
Cigarette smoking	Cigarette smoker (*n* = 22)	51.62 ± 10.92	19 (86.36%)	2 (9.09%)	1 (4.55%)
	Non-cigarette smoker (*n* = 28)	53.04 ± 14.28	21 (75%)	6 (21.43%)	1 (3.57%)
Test of significance		***t =*** 0.379 *P* = 0.707 (NS)	Fisher’s exact test *P* = 0.628 (NS)		
Electronic cigarette smoking	E-cig smoker (*n* = 38)	51.58 ± 11.54	32 (84.21%)^a^	6 (15.79%) ^a^	0 (0%) ^b^
	Non-E-cig smoker (*n* = 12)	55.36 ± 16.94	8 (66.67%) ^a^	2 (16.67%) ^a^	2 (16.67%) ^a^
Test of significance		***t =*** 0.858 *p* = 0.395 (NS)	Fisher’s exact test *p* = 0.049 (S)		
Water pipe smoking	Water pipe smokers (*n* = 34)	53.85 ± 13.86	25 (73.53%)	7 (20.59%)	2 (5.88%)
	Non-water pipe smokers (*n* = 16)	49.5 ± 10.2	15 (93.75%)	1 (6.25%)	0 (0%)
Test of significance		***t =*** −1.114 *p* = 0.271 (NS)	Fisher’s exact test *P* = 0.247 (NS)		

*t*, Student *t*-test of significance; *Sig.*, significant; *S*, significant; *NS*, non-significant; *E-cig*, electronic cigarette

Each subscript letter denotes a subset of group categories whose column proportions do not differ significantly from each other at the .05 level

**Table 4:** Psychiatric symptoms in adolescent smokers according to MINI-KID

		Smoking		OR (95% CI)	Chi-Square test		
		No	Yes				
		*N* (%)	*N* (%)		***χ***^***2***^	*p*-value	Sig.
Depression	No	61 (17.89%)	19 (38%)	0.355 (0.19–0.67)	10.838	0.001	S
	Yes	280 (82.11%)	31 (62%)				
Suicide	No	171 (50.15%)	19 (38%)	1.641 (0.89–3.02)	2.576	0.109	NS
	Yes	170 (49.85%)	31 (62%)				
Mania	No	47 (13.78%)	15 (30%)	0.373 (0.19–0.74)	8.595	0.003	S
	Yes	294 (86.22%)	35 (70%)				
Panic disorder	No	100 (29.33%)	19 (38%)	0.677 (0.36–1.25)	1.55	0.213	NS
	Yes	241 (70.67%)	31 (62%)				
Agoraphobia	No	141 (41.35%)	20 (40%)	1.058 (0.58–1.94)	0.033	0.856	NS
	Yes	200 (58.65%)	30 (60%)				
Separation anxiety	No	84 (24.63%)	16 (32%)	0.695 (0.37–1.32)	1.243	0.265	NS
	Yes	257 (75.37%)	34 (68%)				
Social phobia	No	138 (40.47%)	25 (50%)	0.68 (0.38–1.23)	1.629	0.202	NS
	Yes	203 (59.53%)	25 (50%)				
Specific phobia	No	103 (30.21%)	22 (44%)	0.551 (0.301–1.01)	3.815	0.051	NS
	Yes	238 (69.79%)	28 (56%)				
OCD	No	116 (34.02%)	18 (36%)	0.917 (0.49–1.7)	0.076	0.783	NS
	Yes	225 (65.98%)	32 (64%)				
PTSD	No	225 (65.98%)	31 (62%)	1.189 (0.64–2.2)	0.306	0.58	NS
	Yes	116 (34.02%)	19 (38%)				
ADHD	No	156 (45.75%)	17 (34%)	1.637 (0.88–3.05)	2.44	0.118	NS
	Yes	185 (54.25%)	33 (66%)				
Schizophrenia	No	213 (62.46%)	35 (70%)	0.713 (0.38–1.36)	1.068	0.301	NS
	Yes	128 (37.54%)	15 (30%)				
Binge eating	No	186 (54.55%)	26 (52%)	1.108 (0.61–2.01)	0.114	0.736	NS
	Yes	155 (45.45%)	24 (48%)				
GAD	No	144 (42.23%)	20 (40%)	1.096 (0.6–2.01)	0.089	0.766	NS
	Yes	197 (57.77%)	30 (60%)				

Our study group according to screening questions from The MINI International Neuropsychiatric Interview for Children and Adolescents (MINI-Kid) showed that 90.5% (*n* = 354) experienced mood symptoms at least once as 79.4% (*n* = 311) experienced depressive symptoms and 84.1% (*n* = 329) experienced manic symptoms, while 51.4% (*n* = 201) experienced suicidal ideations at least once, 94.1% (*n* = 368) experienced anxiety symptoms as panic symptoms was experienced by 69.6% (*n* = 272), as well 55.8% (*n* = 218) experienced ADHD symptoms.

### Pattern of smoking

In the study sample, 12.8% (*n* = 50) of the study group were smokers, and 44% (*n* = 22) were conventional cigarette smokers with average age of first exposure to smoke was 13.77±2.37 while the age when the most began to smoke regularly was 15±1.18; 50% (*n* = 11) showed very low severity of smoking (0.7±0.82) and 22% (*n* = 5) showed high severity (5.8±3.35), while 76% (*n* = 38) used e-cigarettes as 39.5% (*n* = 15) were using it for social appearance and 28.9% (*n* = 11) used it to decrease conventional cigarettes use; moreover, 68% (*n* = 34) used water pipe with 79.4% (*n* = 27) showed no dependence (6.3±3.02) while 20.6% (*n* = 7) showed dependence on water pipe smoking (1583±6.52); regarding pattern of smoking 38% (*n*=19) were sole smokers, while 68% (*n* = 31) used more than one tobacco product and there was a significant relation between gender of the adolescent smoker and water pipe smoking as shown in Table 1.

Table 1 shows that there was a significant relation between gender difference and water pipe smoking, while there was no significant relation between socio-economic status and type of smoking

Table 2 shows a significant relation between smoking and impulsivity according to Barratt impulsivity scale.

Table 3 shows that there was a significant relation between electronic cigarette smoking and impulsivity, while there was no significant relation between cigarette smoking and water pipe smoking according to Barratt impulsivity scale 11.

Table 4 shows that there was a significant relation between smoking and depressive and manic symptoms according to MINI-KID

Table 5 shows that there was a significant relation between cigarette smoking, depressive symptoms, manic symptoms, suicidal ideations, panic symptoms, and binge eating symptoms according to MINI-KID**.**

**Table 5:** Psychiatric symptoms in adolescents smoking cigarettes versus other types of smoking according to MINI-KID

Within smokers		cig smoking		OR (95% CI)	Test of significance		
		No	Yes				
		*N* (%)	*N* (%)		***χ***^***2***^	*p*-value	Sig.
Depression	No	18 (64.29%)	1 (4.55%)	37.8 (4.4–324.47)	18.662	<0.001	S
	Yes	10 (35.71%)	21 (95.45%)				
Suicide	No	14 (50%)	5 (22.73%)	3.4 (0.98–11.77)	3.889	0.049	S
	Yes	14 (50%)	17 (77.27%)				
Mania	No	12 (42.86%)	3 (13.64%)	4.75 (1.14–19.83)	5.009	0.025	S
	Yes	16 (57.14%)	19 (86.36%)				
Panic disorder	No	14 (50%)	5 (22.73%)	3.4 (0.98–11.77)	3.889	0.049	S
	Yes	14 (50%)	17 (77.27%)				
ADHD	No	11 (39.29%)	6 (27.27%)	1.73 (0.52–5.77)	0.792	0.373	NS
	Yes	17 (60.71%)	16 (72.73%)				
Binge eating	No	10 (35.71%)	16 (72.73%)	0.21 (0.06–0.7)	6.762	0.009	S
	Yes	18 (64.29%)	6 (27.27%)				
GAD	No	11 (39.29%)	9 (40.91%)	0.93 (0.3–2.92)	0.014	0.907	NS
	Yes	17 (60.71%)	13 (59.09%)				

## Discussion

As tobacco use is alarmingly prevalent among adolescents, it is critical that we understand the implications of use for adolescent health, especially in low- and middle-income countries, where most of the world’s smokers come from. In this study, we explored the health outcomes associated with adolescent tobacco use in Greater Cairo. Results of this study indicate that tobacco use is associated with statistically significant adverse mental health outcomes among adolescents including, depression, mania, panic disorder, binge eating, and suicidality. The magnitude of negative outcomes underscores the urgent need for more focused research in each of these areas to understand the health consequences of adolescent tobacco use in Egypt.

As regards age of the study members, the mean age of the study population in the study was 16.65 ± 0.55 years old that represents the average adolescent age group in Egypt which is 10–19 years old [25]***.***

Considering the size and gender of the study population, our sample size was 391. 55.2% of the study were females while 44.8% were males on contrary to [26] in which 90.4% were males while 9.6% were females but was similar to that of [27] which was 52% females and 48% males, which was quite similar to the actual percentage of adolescent males and females which is 51.2% males and 48.8% females [28].

Regarding the socioeconomic status, our study showed that socioeconomic status showed insignificant relation to tobacco use, a result similar to the one by Sajjadi et al. [29] that showed no significant effect for socioeconomic status on substance use potential including tobacco and that was differed from [30] which revealed that although low socioeconomic status is related to high smoking rates due to lack of social support and poor parenting styles but high socioeconomic status is related to high smoking amounts but this is related for developed countries as in developing countries but we should consider that smoking tobacco is highly prevalent in low- and middle-income countries according to Forouzanfar et al. [13]; our study found that there is no significant relation between socio-economic status and type and pattern of smoking which can be attributed to the availability of all types of smoking with different qualities; also majority of our study sample were of high socio-economic status.

Family history of smoking was significantly related to smoking severity, which was consistent with results by [31, 32]. East et al. [33] considered family smoking as a risk factor for smoking due to observation that normalizes that behavior then imitation and maintenance.

With respect to family history of substance use disorder, about 7.4% had family history of substance use disorder which was significantly related to smoking severity that was consistent with [32] which can be attributed to negligence, gapping between parents and children, and lack of support that led to higher peer influence.

Considering impulsivity in smokers, there was a significant relation between smoking and impulsivity that was consistent with [34] as exposure to smoking in young age increase the liability to impulsive behavior; also, electronic cigarettes smoking in particular showed significant relation with impulsivity, but there was no significant relation between cigarettes and water pipe smoking with it, consistent with [14] that found that electronic cigarettes associated with impulsivity than any other mental disorders, supported by [35] relating tendency of adolescents towards electronic more than conventional cigarettes to novelty seeking to use new forms of tobacco.

Regarding psychiatric symptoms, our study found significant relations between smoking and depressive symptoms and pattern of smoking with simultaneous users of multiple forms of tobacco being more prone for depressive symptoms. Fluharty et al. [36] described the relation between depression and smoking as bidirectional, where smoking tobacco may lead to depression by altering hypothalamic-pituitary-adrenal axis which subsequently causes alteration in the monoamines leading to depression, and depressed patients may use to smoke tobacco aiming to decrease their symptoms through its relaxing effect. This result was consistent with findings by Gorfinkel et al. [37]; however, contested by Farooqui et al. [38] who stated that there is no cause-effect relationship between depressive symptoms and tobacco use and stated that multiple other factors lead to depression.

Another significant relation was found between manic symptoms that was similar to one by Leventhal et al. [14]. Although most studies highlighted the relation between bipolar disorder and cannabis smoking but in our study, we highlighted its relation with tobacco smoking, as it was found that nicotine dependence was associated with neurophysiological changes that increase the vulnerability to mood disorders including manic symptoms [39], supported by Wisnusakti et al. [40] that stated emotional instability, which is very prominent in this age group may contribute in increasing the smoking behavior and severity.

Also, our study found that there is a significant relation between cigarette smoking and binge eating symptoms that was consistent with [7], as high sugar and fat diet triggers the reward system in the same way nicotine causing its dependence [41], which increases the risk for mortality for such patients.

Moreover, we found a significant relation between cigarette smoking and mood symptoms that was consistent with [6] as there is bitemporal relation between them as written before; also our study found a significant relation between cigarette smoking and suicidal ideations and the same was found by Swann et al. [42]; this finding can be attributed to many factors first of them the presence of depressive symptoms which may progress leading to such ideas, also impulsivity which is highly significant with tobacco smoking and negative health consequences of smoking.

Furthermore, it was found that smoking cigarettes is significantly related to panic symptoms and that was consistent with the result by Kunas et al. [43] that found neurobiological overlapping between smoking tobacco and panic disorder.

But our study did not find any significant relation between smoking and obsessive-compulsive disorder which was different from [44]; on the contrary, Jaisoorya et al. [45] have found that obsessive-compulsive disorder is highly significant with smoking which may be attributed to comorbidity with ADHD that was also disagreed with our study that did not find any significant relation between ADHD and smoking unlike Ilbegi et al. [9] that showed that ADHD is highly predictor for nicotine dependence and this can be attributed to underdiagnosis of ADHD in Egypt as most of the parents are seeking psychiatric advice as they are ashamed that their offsprings get diagnosed by a psychiatric diagnosis. Also, our study did not find a significant relation between psychotic symptoms and smoking unlike [6] that may be attributed to those psychotic symptoms affecting cognitive functions, impairing educational performance and attendance, but our study targeted students that attend regularly educational centers and even whom responded to the online survey followed the instructions.

Regarding gender difference and psychiatric symptoms, our study found a significant relation between gender difference, binge eating, and PTSD symptoms in non-smokers. This was consistent with [46] as female adolescents are more prone for psychiatric problems due to unhealthy coping that increase the vulnerability for problematic relations with parents and peers that led to psychiatric disorders alongside with our finding that there is significant relation between gender difference and psychiatric symptoms in smokers with panic symptoms that highlight the relation between smoking and panic symptoms as written before.

Considering prevalence and pattern of smoking, 12.8% of the sample were smokers with 10.65% of females and 15.43% of males being smokers, this was consistent with [47] that found increase in smoking between Arab females including Egypt, but there was a notable difference than the results of [48] where 33.1% of males and 3.3% of females with total about 16.8% of their sample have been smokers. This difference can be attributed to the difficulty to disclose such behavior due to the shameful perception about it that still persists in our society. The percentage was also much less than that found by Mostafa et al. [26] with total smokers about 79.1% of the sample which could be attributed to the sampling method they used targeting adolescents in smoking areas; their study showed similar findings regarding a narrowing in the gender gap in smoking in general. In a study by Hammond et al. [49], the prevalence of cigarette and vaping was about 35% and this could be attributed to the large sample size about 11,800 from 3 countries (England, Canada, USA), also due to a larger acceptability of disclosing smoking behaviors in these societies.

Regarding the form of tobacco use, our study found that 38% were sole smokers and 62% were multiple tobacco form users while Mostafa et al. [26] showed that 55.1% were sole users and 44.9% were multiple tobacco form users.

Mean age of initiating cigarette smoking was found to be 13.77 ± 2.37, while Shalaby and Soliman [50] found initiation to occur at an older age of 18.1 ± 3.1. This difference can be attributed to smokers in university stage while our study is concerned with smokers in secondary stage. Twenty-two (44%) of smokers are using cigarettes more than [26] that found that 37.6% were cigarette smokers; our study found that 22.7% of cigarette smokers have high severity of dependence.

Regarding electronic smoking, our study found that e-cigarette use among students (76%) was more than conventional cigarettes use (44%), a result similar to that by [14] where percentage of electronic cigarette usage (12.4%) was higher than the usage conventional one (4.6 %). This may be due to beliefs that they have fewer negative effects on health regarding medical complications, dependence and bad odor [51], and also variable flavors attracting adolescents [52]***.***

Its availability is more evident than that of conventional cigarettes because of inconsistent restrictions against selling to youth [52] and this was consistent with [35], although it did not report high usage rate for E-cigarettes but it stated high awareness about the E-cigarettes in the younger smokers (42.3%) which can be due to novelty seeking, social media effect and peers’ influence.

Our study found that 39.5% were using it for social appearance similar to [53] that stated that the most common cause was keeping with fashion (33.7%), while in [35], 31.9 % believed that E-cigarettes are less harmful than traditional cigarettes.

Our study also found that (60.5%) of electronic cigarette users are using other types of tobacco. Similar findings in the literature were reported where Pepper et al. [54] found that (66.3%) of respondents usually used e-cigarettes with nicotine; also, Gilbert et al. [55] *stated* that e-cigarettes were very rarely used alone with 93% reported other substance use including conventional cigarettes.

Regarding water pipe smoking, Mostafa et al. [26] showed that 76% of smokers used water pipe, a slightly higher percentage than that we found (66%); however, their results also showed that the majority did not use it on a daily basis. This was consistent with our finding that 79.4% of water pipe users in our sample were no dependent***.*** Widespread use of water pipes between Arab youth despite low dependence related to social factors and shared experiences and to other cues which are specific to this type of smoking as it smells and has bubbling sounds [56].

## Conclusions

Tobacco use is related to statistically significant adverse mental health outcomes among adolescents including, depression, mania, panic disorder, binge eating, and suicidality. The magnitude of negative outcomes underscores the urgent need for more focused research in each of these areas to understand the health consequences of adolescent tobacco use in Egypt.

## Limitation

This study is one of the few to assess a pattern of tobacco use in secondary school students from different areas in Cairo; however, it is not without its limitations. Cairo is mostly made up of urban areas and so the size and location of sample collected make it difficult to generalize the results on all of Egypt, which has both urban and rural populations, which in turn may affect the pattern of smoking. The results from this study serve to explore the pattern of use and warrant an exploration of tobacco use among the adolescent population in other Egyptian governorates.

Accordingly, we recommend surveying larger sample size to get more accurate prevalence of smoking and more accurate correlations, increasing awareness about smoking and its medical complications, and applying steps for restriction of tobacco sales specially for adolescents, with regular scanning for psychiatric symptoms in secondary schools, aiming to start early management and its progression.

## Data Availability

All data generated or analyzed during this study are included in this published article.

## Abbreviations

ADHD: Attention-deficit and hyperactivity disorder
PTSD: Post-traumatic and stress disorder
GAD: Generalized anxiety disorder
OCD: Obsessive compulsive disorder
E-cig.: Electronic cigarettes
S: Significant
NS: Non significant
